# A novel intramedullary callus distraction system for the treatment of femoral bone defects

**DOI:** 10.1007/s11751-016-0255-5

**Published:** 2016-05-24

**Authors:** Konstantin Horas, Reinhard Schnettler, Gerrit Maier, Uwe Horas

**Affiliations:** 1 ANZAC Research Institute, Bone Research Program, University of Sydney, Gate 3 Hospital Road, Concord, NSW 2139 Australia; 2 Laboratory of Experimental Trauma Surgery, Department of Trauma Surgery, Justus-Liebig-University, Rudolf-Buchheim-Str. 7, 35392 Giessen, Germany; 3 Department of Orthopedic and Trauma Surgery, Kliniken des Main-Taunus-Kreises GmbH, Kronberger Str. 36, 65812 Bad Soden, Germany

**Keywords:** Bone defect treatment, Callus distraction, Distraction osteogenesis, Intramedullary, Bone segment transport

## Abstract

An intramedullary device has some advantages over external fixation in callus distraction for bone defect reconstruction. There are difficulties controlling motorized intramedullary devices and monitoring the distraction rate which may lead to poor results. The aim of this study was to design a fully implantable and non-motorized simple distraction nail for the treatment of bone defects. The fully implantable device comprises a tube-in-tube system and a wire pulling mechanism for callus distraction. For the treatment of femoral bone defects, a traction wire, attached to the device at one end, is fixed to the tibial tubercle at its other end. Flexion of the knee joint over a predetermined angle generates a traction force on the wire triggering bone segment transport. This callus distraction system was implanted into the femur of four human cadavers (total 8 femora), and bone segment transport was conducted over 60-mm defects with radiographic monitoring. All bone segments were transported reliably to the docking site. From these preliminary results, we conclude that this callus distraction system offers an alternative to the current intramedullary systems for the treatment of bone defects.

## Introduction

Callus distraction (distraction osteogenesis) is a process enabling the reconstruction of large bone defects and the correction of limb length discrepancies. The principle is the stimulation of new bone formation by creating strain on healing tissue between two bone segments by the application of continuous axial distraction [1]. The two bone segments are generated by a low-energy osteotomy in metaphyseal regions of long bones usually [2]. The technique of creating the osteotomy and the region of the osteotomy are important as the soft tissue envelope and vascularity have to be preserved [3]. For complete regeneration, many interrelated anatomical, biomechanical and biochemical processes must occur in a well-orchestrated manner [4].

Ilizarov described the method of distraction osteogenesis for gradual lengthening of bone using a circular ring fixator [5]. This Ilizarov apparatus is a stable yet dynamic system allowing micromotion and compressive loading at the fracture site promoting callus formation [5, 6]. However, callus distraction using external fixation is associated with problems such as frequent pin-track infections, pain, joint stiffness and axial deviation [7–10]. In an attempt to reduce complications, intramedullary callus distraction systems (IMS) have been developed. Currently, there are several intramedullary devices available [11–14], but few are suited for the treatment of large bone defects [14, 15]. Moreover, these intramedullary devices are associated still with complications such as mechanical failure or pain [16–20]. Consequently, alternative therapeutic approaches such as bone grafting are still used commonly to bridge bone defects; Masquelet et al. [21] described a procedure of combining cancellous autografts with induced membranes that secrete growth factors for stimulating bone regeneration. Although this technique has proven suitable for reconstructing bone defects, it has disadvantages such as a limited supply of bone grafts, morbidity at the donor site (if autografts are used) and nonunion or infection (if allografts are used) [22]. The aim of this study was to design a simple non-motorized intramedullary callus distraction system for the treatment of bone defects.

## Materials and methods

**Fig. 1:**
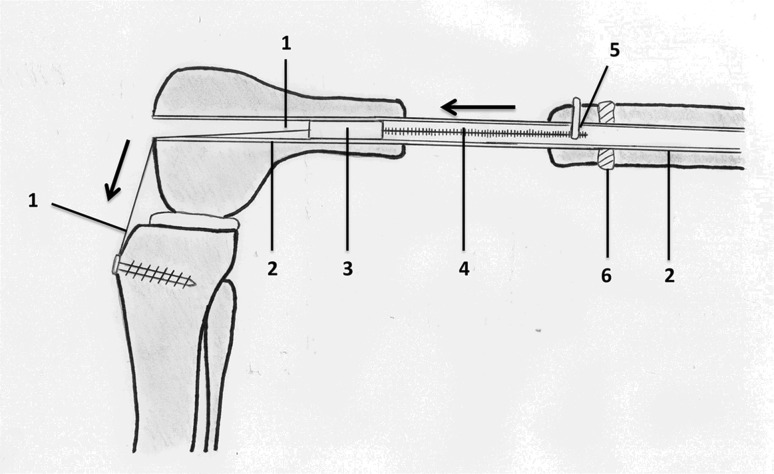
Schematic of CDS implanted into the femur: *1* traction wire fixed to the tibial tubercle, *2* nail, *3* mechanics, *4* threaded rod, *5* spindle nut and connection to bone segment, *6* callus

This novel callus distraction system (CDS) was designed for segmental bone transport in the femur but can be applied to the tibia and humerus also [23]. Distraction osteogenesis is achieved by using a fully implantable system comprising a tube-in-tube system and a wire traction mechanism (Fig. 1). There are three different components enabling a maximum distraction distance of 216 mm for the femoral version of the nail:

1. A locking intramedullary nail
2. The mechanism
3. A traction wire.

### The CDS nail

**Fig. 2:**
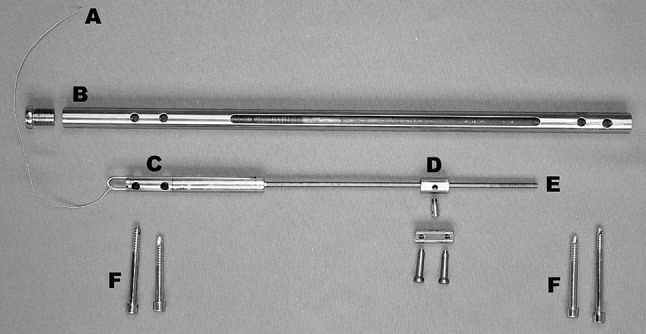
Individual components of the CDS: *a* traction wire, *b* nail, *c* mechanics, *d* spindle nut, *e* threaded rod, *f* interlocking screw

The femoral version of the CDS is a 340- to 420-mm straight nail. It has an external diameter of 13 mm with additional 1-mm longitudinal wall-strengthening bulges leading to a maximum diameter of 14 mm. With an internal diameter of 10.2 mm, the wall thickness measures 1.4 mm (1.9 mm with bulge). To allow transport of a bone segment without rotational deformity, the nail is supported by two proximal and two distal transverse interlocking holes with a diameter of 6 mm each. In addition, there is a 6-mm slit over a length of 216 mm within the nail (Fig. 2).

### Mechanism

**Fig. 3:**
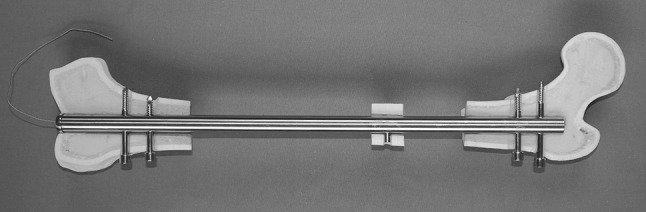
CDS implanted into the femur (anteroposterior view): The traction wire is connected to the fully inserted mechanics

**Fig. 4:**
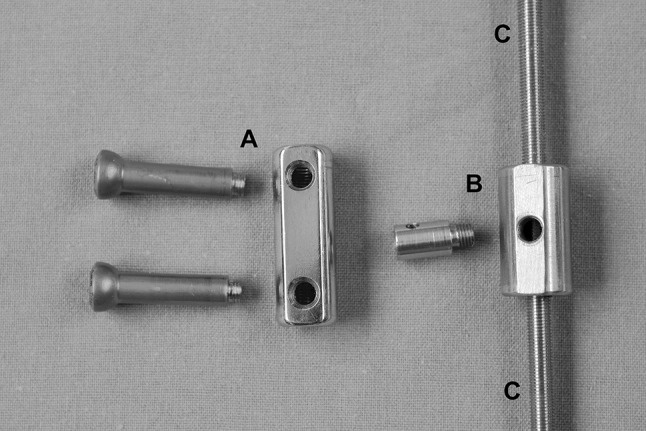
Threaded rod and spindle nut: *a* spindle nut and screws for 6-mm bone segments (screws can also be applied to smaller spindle nut). *b* Spindle nut for 4-mm bone segments. *c* Threaded rod

**Fig. 5:**
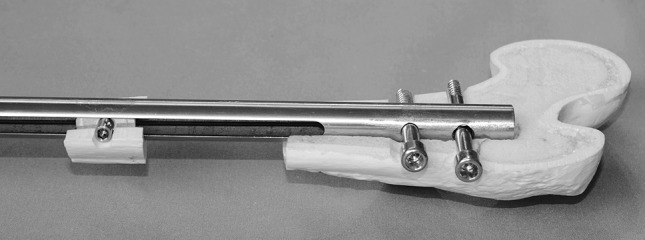
CDS implanted into the femur with bone segment connected to the threaded rod via the spindle nut (lateral view)

With an external diameter of 10.15 mm, the cylinder-shaped mechanical system of the CDS is fully inserted into the nail (Fig. 3). The in-line mechanics consists of a threaded rod and a threaded rod spindle on top. The connection between the bone segment and the threaded rod is produced by a spindle nut attached to the threaded rod (Figs. 4, 5). A traction wire connected to the mechanics creates a force via functional change in the length of the traction wire, occurring on active or passive movement of the knee joint. Movement of the traction wire and tensile force are converted inside the mechanism, which acts in a similar way to a mechanically driven gyroscope, into a rotational movement of the threaded rod which then transports the spindle nut and correspondingly the bone segment connected to the spindle nut. Thus, the mechanism, once set in motion inside the nail’s lumen, turns the threaded rod by converting the translational movement of the traction wire.

### Traction wire

**Fig. 6:**
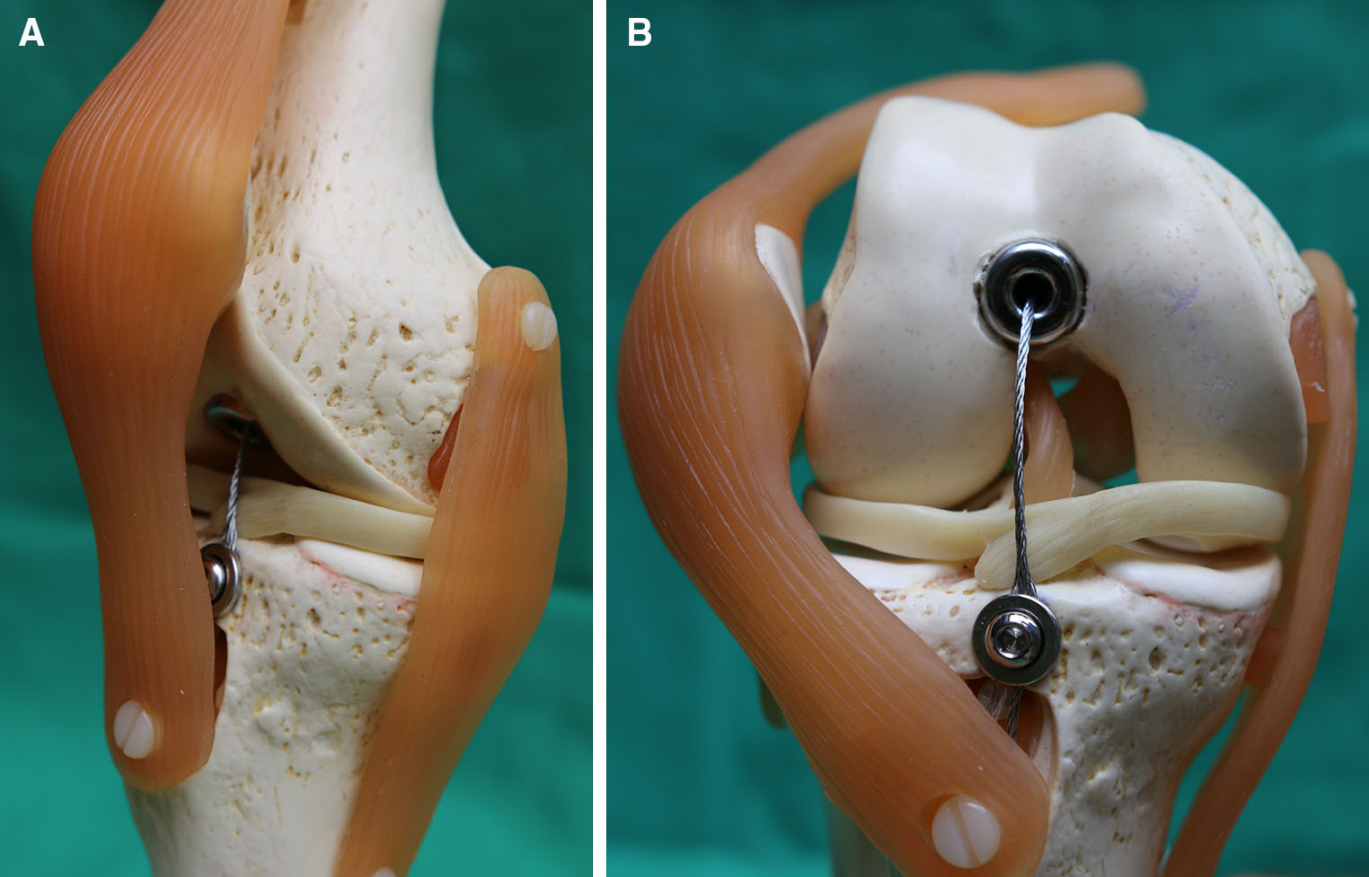
Schematic model of the CDS implanted into the right femur. **a** Antero-medial view at 10° flexion of the knee joint. The traction wire is fixed to the tibial tubercle proximal to the insertion of the patellar ligament. **b** Anterior view at 90° flexion of the knee joint. The patella is laterally dislocated to fully expose the intra-articular running of the wire. The traction wire and the distal end of the nail do not impinge the menisci or impact on the ACL and the retro-patellar cartilage

**Fig. 7:**
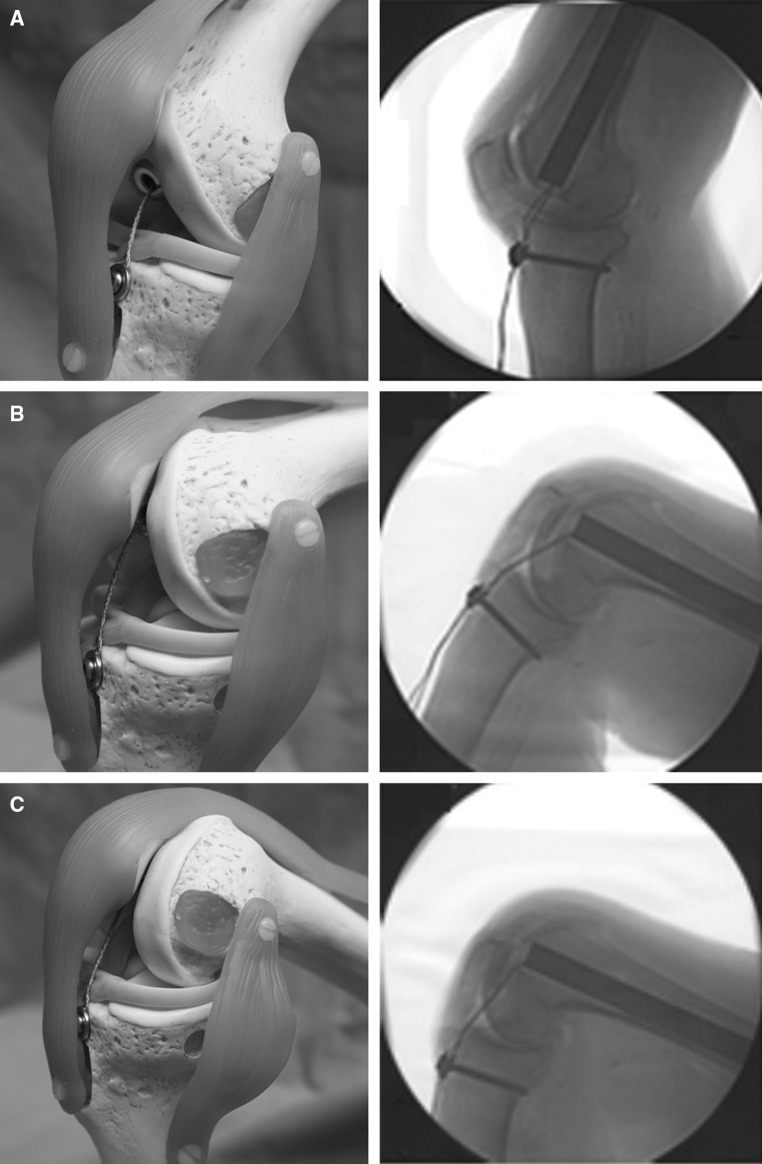
Schematic model of the CDS implanted into the right femur. Antero-medial view at 30° (**a**), 90° (**b**) and 120° (**c**) flexion of the knee joint with correlating X-rays. The length of the intra-articular part of the wire doubled at 120° (**c**) compared to 30° (**a**) having constant traction on the wire

A traction wire is connected to the mechanism on the one end and fixed to the tibial tubercle on the other using a screw as an anchor (Figs. 1, 6). The wire is moved by flexing the knee joint generating a traction force which then triggers the mechanism for bone segment transport. The length of the wire is adjusted at, for example, 90° flexion of the knee joint. Further flexion of the knee joint leads to force transmission as tension is put on the pulling wire (Fig. 7). Knee flexion of more than 120° generates a traction force high enough to trigger the mechanism. The system can be regarded as an all-or-none principle. Flexion of the knee joint from 90° to 119° generates an increasing traction force on the wire, but further traction force is required to release the irreversible bone segment transport. Each flexion of the knee joint over 120° results in a bone segment transport distance of 0.25 mm. It should be noted that the angle that triggers the mechanism is adjustable according to the patient’s range of motion. The designated distance of bone segment transport is 1 mm per day.

### Operative technique and cadaver study

In order to evaluate the implantation of the nail and the system running, a cadaver study using both femora of four human cadavers was conducted. All experiments were approved and conducted in accordance with the guidelines of the Committee of Medical Ethics. Written informed consent from the donor was obtained prior to their inclusion in this study. Each cadaver was thoroughly checked, and none of the cadavers had a history of musculoskeletal disease that could have had an impact on the experiment. All cadavers were frozen to a temperature of −18° Celsius exactly 48 h after death and defrosted for 24 h prior to the experiment. Implantation of the CDS was carried out in supine position using standard retrograde access though the knee joint. The femoral canal was reamed over a guide-wire to a diameter of 15.5 mm followed by temporary insertion of the nail. After removal of the nail, a bone defect was created via a medial approach in order to avoid major damage of the surrounding soft tissues of the femur. Forty-millimeter bone segments on the right femora and 60-mm bone segments on the left femora were generated using a Gigli saw. The osteotomy was performed directly distal to the insertion of adductor brevis in such a way as to preserve as much of the periosteum as possible. The nail was then reinserted into the femoral canal across the bone segment to be transported and then locked proximally in an anteroposterior direction. Standard anteroposterior (AP) and lateral radiographs were obtained to guide the nail to the correct position. Next, a 6.5-mm lateral drill hole was generated on the bone segment followed by fixation of the bone segment on the threaded rod spindle. In order to perform distal locking of the nail, the femur had to be distracted on the side of the osteotomy as the tension force of the adherent soft tissues reduced the initial size of the bone defect. After the size of the bone defect was readjusted to a total length of 60 mm, distal locking was performed and the threaded rod was inserted into the nail. With the mechanism inserted into the nail, the traction wire was adjusted parallel to the anterior cruciate ligament (ACL) and fixed to the tibial tuberosity using a cancellous bone screw (Fig. 6). At completion, the mechanism and the system were tested by flexing the knee joint. Radiographs were taken in AP and lateral direction in order to ensure correct bone segment transport (Fig. 7). Bone transport was then conducted in all eight femora until impingement of the bone segment at the docking site. In clinical application, the screw in the tibial tuberosity will be removed at the end of segment transport and the traction wire will be cut at the distal end of the nail leading to a retraction of the wire into the nail.

## Results

All eight bone segments were transported to the docking site without any complications. During continuous radiographic validation of the CDS, we did not identify any mechanical obstacles of the system or axial deviation. The ratchet system ran smoothly, and no inter-locking of bone segments occurred.

### Implantation

Prior to implantation, each femur was measured and the sizes of the implants were determined. The mean operative time was 75 min (without generation of bone defects). No intraoperative complications or problems occurred. There was no significant relationship, with the numbers available, between height, weight, body mass index (BMI), age of the cadaveric sample and operative time.

### Use in cadavers

The anticipated transport distance of the bone segments was achieved in all eight femora. Bending of the knee joint of more than 120° reliably triggered the mechanism, whereas a knee joint movement between 0° and 119° had no impact. By stretching and flexing the knee joint every 15 s over the entire range of movement (0°–140°), bone transport of the segment over a transport distance of 0.25 mm per cycle was achieved without any difficulty. This procedure was continued until the bone segments had reached the docking site. Radiographs were obtained to evaluate the progress simultaneously showing a consistent pattern. Once the bone fragments had reached the distal segment of the femur, no further passive flexion over 120° of knee flexion was possible. Apart from this, no other passive restrictions in knee movement in the cadavers after implantation of the CDS were noted. Additionally, we examined the intra-articular behavior of the traction wire. Radiographs (AP and lateral) were taken at 0°, 30°, 60°, 90° and 120° flexion of the knee joint (Fig. 7). By flexing the knee joint from 0° to 120°, the length of the intra-articular part of the traction wire doubled compared to its initial length. After passive extension back to the initial position of 0°, no looping of the traction wire occurred (Fig. 7). As the traction wire glided back into the distal end of the nail, no contact of the wire to the menisci or the cruciate ligaments was observed. Notably, movement of the wire occurs inside of the CDS exclusively. As there is always tension on the intra-articular part of the wire, no movement of the wire inside the joint is possible, and therefore, no interaction with the ACL or other soft tissues is to be expected (Fig. 6).

When comparing the sizes of the bone segments (60 and 40 mm) to be transported, no significant difference in implantation or distraction could be found.

### Biomechanics

In order to assess the mechanical stability of the novel CDS, several static and dynamic tests were carried out comparing the novel CDS nail with the Klemm–Schellmann nail [24]. For that purpose a four-point bending test, torsion tests, fatigue tests and a physical check including maximum load testing were conducted. In all tests, material properties showed satisfactory results (Tables 1, 2) and no significant difference compared to the Klemm–Schellmann nail [25–27].

**Table 1:** Experimental data of bending load testing

	Proportional bending moment (Nm)	Stiffness (Nm/angular degree)	Maximal bending moment (Nm)	Bending deformity (angle)
Number of nails tested	10	10	10	10
Mean	83.2	22.1	167.1	14.1
Median	84.0	22.4	168.0	14.1
Standard deviation	5	0.7	3.8	1.3

**Table 2:** Experimental data of torsion stability testing (Nm/°) and prolonged swing testing

	Torsion stability (Nm/angular degree)	Prolonged swing test (load changes)
Number of nails tested	10	4
Mean	0.2930	41,850
Median	0.2965	42,000
Standard deviation	0.00761	1025

Prolonged swing testing was conducted for a period of 60 min and a force of approximately 3 kN at a frequency of 3 Hz. Prior to experiments, we set the threshold to 30.000 load changes calculated based on results by Taylor and coworkers [42, 43]. With a mean value of 41,850 load changes, the novel CDS exceeded the required threshold

### Complications

Difficulties during implantation occurred such as a shortening of the generated bone defect after removal of the bone fragment due to traction forces of the adherent soft tissues. For that purpose, a spacer was inserted and fixed using two pins on subsequent experimental implantation. Nevertheless, this is a problem that only occurs in artificially generated bone defects for the use in this study and does not reflect the situation of bone defect treatment in patients that suffer from bone defects.

## Discussion

The treatment of long bone defects in the lower extremity is a challenging reconstructive problem for orthopedic surgeons. For many years, bone grafting was the most common treatment to bridge segmental bone defects. Since the discovery of distraction osteogenesis, first introduced by Ilizarov, this method has become a successful alternative to bone grafting [5]. This method can be associated with several complications [9, 28]. Problems such as pin-track infection, pain, joint instability and stiffness are related mostly to the external fixator [7, 8, 29]. In an effort to reduce these complications, numerous new devices and implants have been developed [11, 12, 30]. Paley et al compared a standard Ilizarov method to a combination of external fixation with interlocking intramedullary nailing in a study on femoral lengthening. They concluded that lengthening over an intramedullary nail decreases the duration of external fixation, protects against refracture and allows earlier rehabilitation [31]. These results were supported by Kocaoglu et al. [32] in their report of external fixator-assisted bone segment transport over an intramedullary nail for reconstruction of bone defects of the lower extremity. Although several studies showed advantages in combining external with internal fixation, there is, still, the risk of pin-track infection leading to deep intramedullary infection [9, 33, 34]. With fully implantable intramedullary CDS, the potential is to overcome the problem of pin-track infection and to improve comfort during treatment [14, 28]. One of these intramedullary devices is the Albizzia nail comprising two telescopic cylinders in which lengthening is achieved by rotating movements of the limb [11]. Although clinical results were promising, patients complained about pain which made ratcheting difficult [16]. The Intramedullary Skeletal Kinetic Distractor (ISKD) is another mechanically driven device which lengthens through torsional movement of the limb [13]. Several authors published their experience with lengthening using the ISKD and described complications such as runaway nails, premature consolidation, severe pain and uncontrolled lengthening [17–20, 28]. The ISKD has, so far, been described for the use in limb lengthening but not for the treatment of bone defects. Hyodo et al. [30] have recently reported a traction cable device for bone segment transport in the canine femur using an interlocking intramedullary rod for fixation. However, this device comprises an external distraction apparatus, and local infection at the exit side of the cable and along the cable tract has been reported. To our knowledge, only three fully implantable CDS have been described for the treatment of bone defects in humans. The recent Phoenix nail is a magnetically activated drive system, and the first results for the use in bone defect treatment are promising [14]. Another recent development is the magnet-operated telescopic PRECICE nail [35]. It has both CE mark and US FDA clearance for its first- and second-generation implants, and good results for the treatment of limb length discrepancies have been reported [35]. Although the reliability of this novel system seems to be comparable to other intramedullary nails, a magnet-driven device is a novel technology and literature regarding its efficacy, reliability, complication rate and safety is sparse [36]. Baumgart et al. [15] reported a patient with a 12-cm bone defect after tumor resection who was treated successfully using an intramedullary motorized nail (Fitbone). Betz et al. [12] reported also of good clinical outcomes using the Fitbone nail in leg lengthening. These results were further supported by Singh et al. [37] and Krieg et al. [38] who published their experience with the Fitbone nail with a relatively low complication rate of 12.5 % in leg lengthening. Although these devices seem to be appropriate for the treatment of bone defects, few publications exist on their use in bone defect treatment [15]. Moreover, the Fitbone nail comprises a complex motorized mechanism that is expensive and increases the risk of technical failure which further limits its use. For that reason, our aim was to design a simple and non-motorized intramedullary CDS as a reasonable alternative to the currently existing treatment options.

In this study, we introduce a fully implantable CDS for the treatment of femoral bone defects. This novel intramedullary callus distraction system was subjected to several mechanical tests and a cadaver study with promising results. In our cadaver experiment, bone segment transport was accomplished without mechanical obstacles and the desired range of motion of the knee joint achieved. A major advantage of this CDS is that it allows physiological movement of the limb and helps prevent the frequently reported complication of knee joint contracture [9]. It is inevitable that some movement is lost, albeit temporarily, during the period of bone segment transport. However, this limitation can be minimized by adjusting the traction wire according to the patients’ knee movement range. For example, if the traction wire is adjusted such that the mechanism is triggered by flexing the knee joint more than 120°, any movement between 0° and 119° is possible without any effect on the mechanism. At the end of transport, the wire and screw will be removed and further bending of the knee joint is possible without any restriction. Other mechanical devices either limit knee movement range or require frequent non-physiological and painful movement; in this novel CDS, only four cycles of knee flexion are necessary to reach the designated transport distance of 1 mm per day compared to 15 cycles of rotational movement of the femur using the Albizzia nail. An important consideration is that the data presented in this study are from an experimental setup; there are limitations on transferring the results into clinical practice. For the present time, this study confirms proof of concept that the mechanism designed for the purpose of bone segment transport within an intramedullary nail works.

There are different opinions on the adequate velocity of distraction in order to prevent premature consolidation [18, 19, 29, 39]. The velocity of distraction in this CDS has the potential to be adjusted by the patient facilitating a personalized distraction rate. Nevertheless, as with all other systems used in distraction osteogenesis, good compliance and understanding by the patient is mandatory for success. Another factor that should be taken into consideration is that the nail is designed for weight bearing (at least at an axial load of 20 kg which corresponds clinically to partial weight bearing). Axial micromotion and compressive stress at the fracture site are considered beneficial for bone healing [40, 41], and therefore, the period of time to full consolidation of the regenerated bone might be reduced in this system.

## Conclusion

The findings of this study demonstrate the feasibility of bone segment transport by callus distraction using a novel CDS. Results achieved in mechanical experiments and in the cadaver study provide proof of concept that the mechanism designed is able to transport a segment of bone in the femur. These initial results have to be validated further and the novel CDS was introduced in animal experiments.
